# Early Recognition and Risk Stratification in Cardiogenic Shock: Well Begun Is Half Done

**DOI:** 10.3390/jcm12072643

**Published:** 2023-04-01

**Authors:** Effie Polyzogopoulou, Sofia Bezati, Grigoris Karamasis, Antonios Boultadakis, John Parissis

**Affiliations:** 1 Emergency Department, Attikon University Hospital, National and Kapodistrian University of Athens, Rimini 1, Chaidari, 12462 Athens, Greece; 2 Second Department of Cardiology, Medical School, Attikon University Hospital, National and Kapodistrian University of Athens, 12462 Athens, Greece

**Keywords:** cardiogenic shock, risk stratification, risk scores, management, shock team, shock network

## Abstract

Cardiogenic shock is a complex syndrome manifesting with distinct phenotypes depending on the severity of the primary cardiac insult and the underlying status. As long as therapeutic interventions fail to divert its unopposed rapid evolution, poor outcomes will continue challenging health care systems. Thus, early recognition in the emergency setting is a priority, in order to avoid delays in appropriate management and to ensure immediate initial stabilization. Since advanced therapeutic strategies and specialized shock centers may provide beneficial support, it seems that directing patients towards the recently described shock network may improve survival rates. A multidisciplinary approach strategy commands the interconnections between the strategic role of the ED in affiliation with cardiac shock centers. This review outlines critical features of early recognition and initial therapeutic management, as well as the utility of diagnostic tools and risk stratification models regarding the facilitation of patient trajectories through the shock network. Further, it proposes the implementation of precise criteria for shock team activation and the establishment of definite exclusion criteria for streaming the right patient to the right place at the right time.

## 1. Introduction

Cardiogenic shock (CS) represents a life-threatening condition equated to a dismal prognosis [1]. Since the introduction of the fundamental mechanisms of shock in 1972 [2], CS has been universally defined as a state of severe end-organ hypoperfusion and tissue hypoxia resulting primarily from cardiac pump failure [1,3,4].

Epidemiological data over the last decade report a significant increase in the incidence rate of hospitalizations, while in-hospital mortality has not shown any significant improvement [5,6,7]. Traditionally, acute coronary syndrome (ACS) has been the leading cause of CS, accounting for approximately 80% of cases [8]. However, recent observational studies report a declining incidence of ACS-CS, ranging from 30 to 55.4% [6,7,9,10], with a concurrent increase in the incidence of CS of other etiologies, mainly decompensated heart failure (AHF-CS). With the advent of the early revascularization strategy [11,12] established by the SHOCK trial [11], mortality rates significantly decreased from 80% [13] to 40–50% [14] in the late 1990s and early 2000s. However, in the last 15 years, CS mortality has reached a plateau even in the most leading-edge cardiac centers, with estimates of approximately 30–40% [6,10,15]. The proven efficiency of standardized protocols through organized structures such as catheterization laboratories and trauma centers for the management of ST elevation myocardial infarction (STEMI) [16] and polytrauma [17] patients, respectively, suggested that the development of cardiac shock centers and the establishment of a prespecified patient pathway could ideally contribute to an improvement in CS survival [11]. Considering the significant percentage of patients presenting with CS in the emergency department (ED) (15% of patients with shock) [18] along with the high ED mortality of severe heart failure patients [12], emergency physicians play a pivotal role in the chain of survival of patients presenting with cardiogenic shock in the ED. A prompt and structured approach in the ED can maximize the likelihood of a favorable outcome for those patients. The ED’s contribution is focused on early detection as well as timely referral to advanced cardiac care centers.

Apart from the optimization of care systems, a thorough insight into the complexity of the syndrome is essential to overcome challenging issues in the management of CS patients. The intricate relationships among various etiologies, different hemodynamic phenotypes and heterogeneous patient populations underscore the need for a more concrete classification and risk stratification in order to improve outcomes [1]. In addition, study design protocols mandate the establishment of uniform criteria, applicable at bedside, to tailor pharmacological and mechanical interventions and assess patient response.

The aim of this review is to focus on the initial approach to CS, pointing out the pivotal role of the ED for the early identification and optimal initial management of CS, and to summarize current evidence on practical handling issues. Additionally, it highlights the importance of a multidisciplinary approach through the shock team, and it defines the criteria for shock team activation as well as criteria of exclusion for advanced treatment.

## 2. Early Recognition

### Cardiogenic Shock Definition and Severity Classification

Clearly defined criteria are usually the mainstay of early recognition in clinical medicine. Traditionally, in clinical trials, cardiogenic shock diagnosis has been based on the presence of hypotension (systolic blood pressure (SBP) < 90 mmHg for >30 min or the need for pharmacological or mechanical support to maintain SPB > 90 mmHg), along with end-organ hypoperfusion (altered mental status or cool extremities or lactate > 2.0 mmol/L or urine output < 30 mL/h). In addition, pulmonary congestion, or invasive hemodynamic criteria [Cardiac Index (CI) ≤ 2.2 L/min/m^2^ and pulmonary capillary wedge pressure (PCWP) ≥ 15 mmHg] have been used [19]. The latest guidelines of the European Society of Cardiology (ESC) for the diagnosis and treatment of acute and chronic heart failure [4] focus on the presence of hypoperfusion based on clinical signs (such as cold and sweaty extremities, oliguria, altered mental status, dizziness, narrow pulse pressure) and on addition laboratory parameters (such as elevated creatinine, metabolic acidosis, and elevated serum lactate). Importantly, it is acknowledged that shock may be present even without hypotension [20]. Normotensive shock may be manifested due to increased vascular resistance as a compensatory mechanism of counterbalancing reduced cardiac output. Recent studies highlight the importance of identifying CS in the initial stages, given the increased mortality of patients with hypoperfusion in the absence of hypotension, compared to patients with isolated hypotension [21].

The principal limitation regarding the aforementioned criteria is that CS is approached as a binary condition. However, CS in clinical practice does not represent a static state but comprises a dynamic process with a spectrum of clinical presentations, alternating through various degrees of severity, time progression or response to therapy. Thus, in 2019, the Society for Cardiovascular Angiography and Interventions (SCAI) proposed a shock severity classification which covered the relevant practical gap in the formerly used CS criteria and bedside clinical assessment. CS patients were categorized into five clinical stages: A (at risk) for developing CS, but hemodynamically stable; B (beginning), clinical evidence of hemodynamic instability determined by tachycardia (heart rate > 100 beats per minute) or hypotension (SBP < 90 mmHg, MAP < 60 mmHg or >30 mmHg drop from baseline), but without hypoperfusion; C (classic), clinical and biochemical evidence of hypoperfusion that requires pharmacological or mechanical support, usually, but not always, accompanied by hypotension; D (deteriorating), clinical evidence of shock (as in stage C) and failure of the initial support strategy to restore perfusion as evidenced by worsening hemodynamics or rising lactate; E (extremis), refractory shock or circulatory collapse with highly deranged biochemical markers (lactate > 8 mmol/L, pH < 7.2, base deficit > 10 mEq/L). Cardiac arrest is cited as the “A modifier” that aggravates clinical outcomes, regardless of stage classification [22].

Since its publication, several observational studies have validated the SCAI SHOCK classification in various populations and clinical settings [23]: patients with AMI-CS [24,25,26] and HF-CS [25,26,27,28,29], and patients with out-of-hospital cardiac arrest (OHCA) [25,26,30,31]. All studies found a correlation between SCAI SHOCK stage and mortality, revealing increasing mortality rates in more advanced stages of shock severity. Successively, the SCAI expert consensus writing group proposed a three-axis model of risk stratification, considering shock severity, phenotype and risk modifiers, and recommended a more individualized approach [23]. Lately, the SCAI CS working group presented a novel algorithm to enhance the application of the SCAI SHOCK classification on the basis of distinct clinical parameters, with defined cut-off values for each SCAI stage; parameters included OHCA, serum lactate (≤2, 2–5, or >10 mmol/L), ALT (≤200, 200–500, or >500 IU/L), SBP(<60, 60–90, >90 mmHg) and pH (≤7.2 or >7.2). In addition, the study group identified a significant association between baseline stage and in-hospital mortality and poorer prognosis for AMI-CS with respect to HF-CS patients and for those experiencing out-of-hospital cardiac arrest (OHCA) or receiving intensified treatment [32].

Table 1 outlines the definitions most widely used.

## 3. Initial Diagnostic Approach

CS management mandates a short timeframe, widely referred to as the “golden hour” [33]. Prompt identification following a comprehensive assessment will allow timely resuscitation and early management/disposition decisions [4]. For patients not already hospitalized, the emergency physician plays the critical role of within-hospital first medical contact, and her/his actions will determine patient outcomes to a significant degree. For patients with CS, the “door to balloon” time used for patients with STEMI requiring primary percutaneous coronary intervention (PCI) has been translated into “door to support” [34].

### 3.1. Detection of CS

An initial systematic Airway, Breathing, Circulation, Disability, Exposure (ABCDE) approach ensures prompt and holistic evaluation of patients with suspected CS [35]. Close monitoring of blood pressure, heart and respiratory rate and oxygen saturation is required for continuous assessment of clinical status, disease severity and response to treatment [1,3,4]. The main goals in the primary investigation are the identification of signs of hemodynamic compromise and the assessment of end-organ perfusion and volume status. Hemodynamic instability can manifest as hypotension ((SBP) < 90 mmHg for >30 min or the need for pharmacological or mechanical support to maintain SPB > 90 mmHg) or tachycardia (HR > 100 bpm). As shock is characterized by overt catecholamine activation with subsequent vasoconstriction, relative hypotension and narrow pulse pressure are high risk signs that demand meticulous evaluation [1,3,36]. Additionally, the diagnosis of shock demands the presence of hypoperfusion, which needs to be identified early by the determination of critical signs such as altered mental status, cold and sweaty extremities, prolonged capillary refill time (>2 s) and oliguria (<30 mL/h).

In parallel, the respiratory status necessitates early evaluation and management and characterizes the patient phenotypic profile. Physical findings of orthopnea, tachypnea, hypoxemia, cyanosis, lung crackles and respiratory wheezing are signs of respiratory distress and volume overload.

The Forrester classification [37] of patients as “cold and wet” (classic CS), “cold and dry” (euvolemic CS), and “warm and wet” (vasodilatory CS) [3] using the aforementioned clinical criteria may assist in the identification of cardiac involvement (left- versus right-sided or biventricular failure) and in the guidance of initial therapy and development of a treatment plan through the cardiac shock network. Routine invasive hemodynamic evaluation with a pulmonary artery catheter (PAC), measuring CI and PCWP, has an ambiguous contribution to mortality [38,39], and due to its difficult applicability in the ED environment, it is only recommended in patients with CS of unknown etiology or with refractory CS, who need mechanical circulatory support [40,41].

Cardinal signs and findings that may aid in the diagnosis and phenotyping of CS are diaphoresis, lower limb edema, hepatomegaly, jugular distension and the presence of heart murmurs.

### 3.2. Detection of CS Etiology

The prompt comprehensive diagnostic approach facilitates the identification of the CS cause. Identifying the underlying cause early is critical, as the patient’s subsequent management should be targeted and can be significantly different depending on etiology. The principal element of the primary evaluation is the awareness and investigation of potential causes of circulatory compromise [1,3,4]. Practitioners should follow a focused algorithm denoted by the CHAMPIT acronym (C indicating ACS; H, Hypertension emergency; A, Arrhythmia; M, Mechanical cause; P, Pulmonary embolism; I, Infection; and T, cardiac Tamponade) [4]. Reversible causes of CS necessitate prompt identification and management, particularly ACS whose prognosis is significantly correlated with an immediate reperfusion strategy [13]. Similarly, patients should be assigned for surgical repair in cases of mechanical complications of ACS, valvular causes or aortic dissection, and optimal medical management should be provided early in the course of HF-CS of alternative etiologies (acute or decompensated HF, arrhythmias, myocarditis, Takotsubo cardiomyopathy, infection) [4]. Furthermore, appropriate MCS should be considered early for patient stabilization before definite treatment, as a bridge to decision, bridge to bridge or bridge to recovery, in relation to the etiological trigger [1]. Valuable bedside diagnostic examinations for the immediate detection of the CS cause include electrocardiograms (ECG) and ultrasound, while blood gas analysis and biochemical studies may contribute to the holistic assessment of CS patients.

#### 3.2.1. ECG

Upon patient presentation, a 12-lead electrocardiogram is mandatory due to its diagnostic role in the identification of tachy- or bradyarrhythmias and ACS (ST or non-ST- segment elevation myocardial infraction) and emergent activation of the cardiac catheterization laboratory when needed [1,3,42]. In patients presenting with chest pain, identification of AMI should be undertaken without delay in order to activate their direct transfer to PCI-capable centers. Therefore, ECG should be carried out in the pre-hospital setting, at fist medical contact by the emergency medical system (EMS). Furthermore, ECG interpretation may reveal characteristic features of other causes of CS such as myocarditis, Takotsubo cardiomyopathy, pulmonary embolism, cardiac tamponade and electrolyte abnormalities [3].

#### 3.2.2. Ultrasound

Echocardiography is highly recommended as part of the initial approach for CS patients. Prior to a comprehensive assessment of cardiac performance, point-of-care ultrasound (POCUS) should be performed, applying standardized and restricted protocols primarily in order to distinguish CS from other shock types, assess cardiac contractility, check for the presence of congestion, and identify CS etiology [1,43]. In a systematic review, POCUS enhanced the diagnostic performance of patients with undifferentiated shock and improved etiological diagnostic certainty of CS patients, compared to clinical examination without the use of POCUS [44]. Various POCUS protocols, such as the RUSH (Rapid Ultrasound in Shock) protocol, are used widely in order to differentiate shock types (obstructive, distributive, hypovolemic or cardiogenic) and to identify shock etiology and physiopathology. Focused evaluation involves estimation of “the pump” (left and right ventricular function and sizes, accumulation of pericardial fluid) and “the tank” (inferior vena cava (IVC) dimension and collapsibility, lung congestion indicated by the presence of B-lines and pleural effusion, free abdominal fluid and signs of tension pneumothorax) and finally, assessment of “the pipes” (vascular system: aorta, femoral and popliteal veins) [45]. Recently, another protocol has been proposed for the early identification of CS in patients presenting in the ED with undifferentiated hypotension, based on semi-automated imaging software (SAIS). ED physicians were able to obtain advanced measurements of hemodynamic compromise, such as LVOT VTI (left ventricular outflow tract velocity time integral) in relation to IVC collapsibility and B-line pattern, and identify patients at risk for CS [46].

As soon as CS is identified as the most plausible type of shock, a more thorough echocardiographic investigation should be conducted with the aim to confirm the diagnosis and to investigate the presence of mechanical complications of AMI or severe valve dysfunction. Moreover, it may aid in the identification of candidates for MCS and subsequent device positioning [1,47].

Further, bedside echocardiographic monitoring may provide essential information regarding hemodynamic status, response to fluid challenge, and pharmacological and mechanical interventions (IVC respiratory variation and collapsibility index; B-line lung pattern; LV and RV contractility and diastolic diameters; LV filling pressure (e/e’) and RV filling pressure). Echocardiography should be combined with venous ultrasound (hepatic, portal and intrarenal veins) so as to assess venous congestion as a marker of organ congestion and damage [47,48].

#### 3.2.3. Blood Gas Analysis

Serum lactate measurement on admission is recommended as a marker of hypoperfusion and tissue hypoxia [49,50]. A cut-off level of 2 mmol/L has been suggested by the SCAI SHOCK workgroup [22] to support CS diagnosis. Additionally, serial lactate measurements are a useful tool in order to estimate a patient’s response to therapy [51] and prognosis [21]. Recent studies underline the importance of lactate clearance (*LC*) as a prognostic marker, superior to baseline lactate, since higher *LC* has been significantly associated with better prognosis [52,53]. Lactate clearance is the reduction in lactate concentrations at two different timepoints and can be measured by calculating the percentage of the exact time difference (Δ*t*) between lactate concentration on admission (*L*1) and lactate concentration after initial resuscitation (*L*2) [53].

(1) $$LC(\%/h)=\frac{L1-L2}{L1*\Delta t\left(L1,L2\right)}*100$$

(*L*1 and *L*2 are prespecified time points).

In the context of established CS, arterial blood gas analysis may result in findings of metabolic acidosis with hyperlactatemia and increased base deficit [54]. pH and base deficit have been used as markers of shock severity in various risk/severity models [19]. Furthermore, ABG is used to assess features of respiratory failure with low partial oxygen pressure or respiratory acidosis [1].

#### 3.2.4. Laboratory Evaluation

Troponin and natriuretic peptides are additional biomarkers of diagnostic and prognostic importance and are useful in the identification of etiology (AMI versus HF), as they may reflect myocardial necrosis and cardiac wall stress, respectively; however, pending results should not delay initiation of pharmacological and revascularization therapy [1,3,55]. Point-of-care troponin tests may offer valuable information in the emergency setting, allow the early identification of ACS and differentiate CS from other causes of shock [56]. Several novel biomarkers seem to have a promising prognostic role in patients with acute heart failure and may contribute to patient risk stratification. Bioactive adrenomedullin (bio-ADM) may aid in the early detection and severity assessment of congestion [57], and proenkephalin A may be helpful in predicting worsening renal function in acute heart failure patients [58]. Both have a recognized role as prognostic markers of mortality [59,60]. Additionally, growth differentiation factor-15 (GDF-15) [61], associated with upregulation of inflammatory pathways, and angiopoietin-2 [62], related to vascular instability, were independently associated with poor short- [61] and long-term mortality and reperfusion success [62] in AMI-CS patients, in two subanalyses of the IABP-SHOCK II Trial.

Other important laboratory investigations include renal and liver function tests, coagulation studies and serum electrolytes [1,3]. Acute kidney injury complicates 15–55% of patients with CS and is strongly correlated with increased mortality [63,64]. Serum creatinine and urinary output are useful indicators of renal perfusion and escalation of therapy with renal replacement therapy (RRT). Moreover, levels of alanine transaminase (ALT), aspartase transaminase (AST), alkaline phosphatase (AP), serum bilirubin and lactate dehydrogenase should be monitored for evidence of hepatic injury and attributed to either liver congestion or ischemia [65]. A cut-off level of ALT > 200 U/L has been lately proposed as a clinical criterion of shock severity according to the SCAI SHOCK staging [32].

## 4. CS Risk Stratification and Risk Scores

As already discussed, CS extends not only across a wide range of causes, but also across a wide spectrum of clinical presentation, severity, and prognosis. This is one of the reasons why clinical trials that treat CS patients as homogenous cohorts often come back negative. The use of temporary mechanical circulatory support (t-MCS) is such an example. International guidelines suggest that t-MCS should be considered, but the patients who will benefit are not clearly described, nor are the cases in which escalating care would be futile. The task of patient selection for therapy escalation becomes even more difficult due to the time-sensitive course of CS; decision should be taken early, and interventions should take place ideally before irreversible damage is established. Continuous clinical examination and serial review of shock severity stage allows for early identification of deterioration implying the need for escalation of therapy [22]. The challenge with CS management is individualizing care as much as possible, as the strategy of “one size fits all” (e.g., IABP for all, or no use of IABP at all) has failed. Therefore, the estimation of an individual patient’s prognosis is essential in order to expedite decision making: patient at risk of deterioration, so extra monitoring/management care needed; need for escalation to MCS; futility of further escalation; decision to de-escalate. Furthermore, standardized risk prognostication facilitates communication among physicians and medical groups who are responsible for patient management (e.g., ED physician and interventional cardiologist, ED team and ICU). Additionally, it allows communication with the patients’ next-of-kin/family on a realistic and honest basis. Finally, it is required to conduct clinical studies with tailored therapies and increase comparability of different studies. Several scores for patient risk stratification have been suggested to reinforce physicians’ decision making with tools for outcome prognostication and therapeutic plan organization. However, despite the abundancy of available risk scores, their use remains limited in the acute setting, as they derive from intensive care unit (ICU)/cardiac ICU registries or from AMI-CS populations. Moreover, they entail differences in the definition of CS (hemodynamic versus clinical criteria) and in therapies provided (pharmacological versus mechanical support).

The majority of risk scores stem from AMI-CS registries [66,67,68]: the ORBI score [66], SHOCK score [67] and IABP-SHOCK-II score [68] have high predictive value for mortality [67,68] or predict the probability for CS after PCI following AMI [66]. The growing need to address mixed populations of CS has been appraised in the CardShock [8] and the INOVA [69] scores. Although the variables in the CardShock score are easy to estimate on presentation, the score does not incorporate the potential need for MCS or provide prognosis for short-term mortality [8]. On the other hand, the INOVA score is applicable only for cardiac intensive care unit patients invasively monitored with PAC [69]. The IABP-SHOCK-II and CardShock scores have received external validation, with modest predictive value for non-AMI-CS patients [70]. The SCAI SHOCK classification is a valuable score for evaluating disease severity of various CS etiologies and through serial time points and clinical care settings, but it has only been validated retrospectively [32]. However, its use is appealing due to its feasibility in the ED setting and its ability to estimate clinical status at bedside. A thorough review of the abundancy of risk scores developed over the last five decades underscores the necessity of a novel risk score applicable at bedside and on admission, so as not only to predict mortality but also to promptly identify patients who will probably benefit from advanced therapies and to contribute to decision making [71].

Recently, a novel risk score has been published, the CSP (Cardiogenic Shock Prognosis), based on retrospective data from patients presenting at the ED with CS of various etiologies. A nomogram was established incorporating information from a patient’s medical history, point-of-care and laboratory testing and medical interventions made within 72 h of admission. The score proved to be a reliable predictive tool for in-hospital mortality, but external validation studies are needed [72].

A novel risk model that could anticipate the diagnosis of CS two hours before its development has been proposed lately by Chang et al. The authors proposed an algorithm based on a machine learning model that identified older age, male gender, higher troponin level, lower pulse pressure, medium level of immature granulocytes, higher O_2_ saturation, and lower bicarbonate as risk factors that, in correlation with the clinical picture, could alert physicians to the increased probability of a patient’s entering the lethal spiral of CS, or likewise stages A or B of the SCAI SHOCK classification [73].

Interestingly, a simple score for the identification of cardiogenic hypotension in the ED has been described, evaluating findings integrated in an emergency assessment. High troponin levels, ECG signs of ischemia, shortness of breath, absence of fever and history of heart failure were independent predictive factors of cardiogenic hypotension. A score ≥ 2 has shown 82% sensitivity and 75% specificity in identifying CS in the ED [74].

A different approach in the evolution of risk stratification models is based on biomarker criteria. The CLIP score, designated in a population of AMI-CS patients, considers the complexity of CS irrespectively of hemodynamic measurements. With a view of the deranged systemic pathways following the primary cardiac insult, it evaluates levels of cystatin C, lactate, interleukin-6 (IL-6) and N-terminal pro-B-type natriuretic peptide as markers of impaired renal function, hypoperfusion, inflammation and congestion, respectively. The score was reliably predictive of mortality and externally validated but only in AMI-CS patients [75].

Table 2 summarizes the most useful risk stratification scores.

## 5. Initial Management

Current guidelines [1,4] set a number of goals at first medical contact: symptom alleviation, organ perfusion and congestion improvement, oxygenation support and organ damage limitation.

### 5.1. Symptom Relief

Pain and anxiety should be cautiously managed with the administration of morphine in selected patients with intense insisting pain not resolving with supportive treatment. Routine use of opiates is not recommended [4], as morphine has been associated with increased need for mechanical ventilation support, prolonged hospitalization and worse prognosis [76].

### 5.2. Fluid Resuscitation

In the absence of signs of congestion and in patients with preload-dependent phenotypes, fluid resuscitation should be considered with boluses of normal saline or Ringer’s lactate, 250 mL over 15–30 min and under close monitoring with POCUS [1]. POCUS is a dynamic tool for the real-time and serial assessment of volume status and systematic congestion in order detect volume overload and to guide fluid administration. Valuable parameters for optimizing and monitoring fluid resuscitation include the IVC respiratory variation and collapsibility index, the presence of a B-line profile in the lungs, LV and RV diastolic diameters, LV filling pressure (e/e’) and RV filling pressure [1,43], as well as VExUS score [48]. Passive leg raising (PLR) could be reliably used in order to assess fluid responsiveness [77,78]. Fluid responsiveness is the increase in cardiac output of greater than 10% following a 500 mL fluid bolus [79]. PLR, acting as an endogenous fluid challenge, augments venous return, central venous pressure and biventricular preload, and the eventual rise in cardiac output indicates the need for volume expansion. Performance of the test in the ED setting could be aided by the use of echocardiography, estimating changes in the CO through the measurement of the left ventricular outflow tract velocity time integral (LVOT VTI) [80,81].

Currently, data on crystalloid type remain controversial for use in sepsis and shock. There is lack of robust data in the literature specifying the appropriate type of crystalloid fluid for CS patient resuscitation. There is a relative concern regarding the use of saline solutions, as they may cause hyperchloremic metabolic acidosis and acute kidney injury (AKI) [82], and since cardiorenal syndrome is a common complication of CS [83], selection of the least harmful fluid retains a significant importance. Studies on critically ill ICU patients have shown a favorable effect of balanced crystalloid (plasmalyte or Ringer’s lactate) versus saline administration with respect to the need for renal replacement therapy (RRT), AKI and mortality [84,85]. Hammond et al. showed in a metanalysis that administration of balanced crystalloids resulted in a relative reduction in the risk of death at 90 days, ranging from a 9% to 1% relative increase [86]. However, two other metanalyses concluded that there was no statistically significant difference between administration of balanced crystalloid solutions and saline in terms of mortality, incidence of AKI and RRT [87,88]. Given the rather contradictory results, and as critically ill ICU patients represent fairly heterogeneous populations, more studies are needed in order to elucidate which is the appropriate fluid therapy for such high-risk patients, especially those with CS.

### 5.3. Oxygenation Support

The need for immediate intubation and mechanical ventilatory support must be addressed on arrival on a patient-to-patient basis [4]. Clinical presentation and point-of-care-acquired data will define the choice of noninvasive ventilatory support (NIV) versus invasive mechanical ventilatory support (IMV) [89]. The majority of patients with signs of congestion and without signs of acute RV failure will benefit from positive pressure ventilation both in respiratory and hemodynamic features, since the mechanical-ventilation-related decrease in left ventricular (LV) preload and afterload can reduce the workload of the failing LV [90,91]. Special caution must be taken with hemodynamically unstable patients who are not responding to initial therapy with vasopressors and inotropes and with those with signs of acute RV failure, where the reduction in the preload of the RV and increase in pulmonary vascular resistance caused by positive intrathoracic pressures may lead to further hemodynamic deterioration [8,91]. In this context, in most of the patients with CS, an early NIV trial of 30 min to 1 h will be beneficial, along with initial hemodynamic stabilization, and may help to avoid endotracheal-intubation-related risks such as airway complications, further destabilization due to anesthesia induction and the need for ongoing sedation [90]. Furthermore, IMV is associated with complications such as ventilator-acquired pneumonia, increased length of ICU stays, and increased in-hospital mortality [92]. Major contraindications to NIV are altered mental status on presentation, refractory hypotension, acute RV failure, facial deformities, secretions and vomiting, as well as an uncooperative patient [8,91,93]. The choice of NIV mode (C-PAP vs. Bi-PAP) depends mainly on the presence of hypercapnia, which outlines the need to support both oxygenation and ventilation [89]. Nevertheless, in patients scheduled for primary coronary intervention, the choice of IMV with early intubation and stabilization during the ED stay may be preferential, considering patient control, safety and positioning in the cath-lab. The indications and initial settings for both NIV and IMV are presented in Table 3.

### 5.4. Vasoactive Agents

Inotropes and vasopressors represent a necessary evil in the initial management of patients on the verge of circulatory compromise. They are indispensable and promptly available pharmacological agents, but they should be used at the lowest possible dose and for the lowest possible duration [94,95,96], since prolonged administration is associated with increased oxygen demand (further aggravating myocardial ischemia), increased afterload, impaired microcirculation, arrhythmogenesis [97] and mortality [98]. Their hemodynamic effects may vary, and selection of the appropriate agent should be based on CS etiology, hemodynamic profile and shock severity [22,97,99].

Use of vasopressors and inotropes should be individualized based on patient fluid status and CS etiology and should be adjusted based on clinical judgement. In fluid-responsive patients with signs of hypoperfusion (tachycardia and vasoconstriction) but without hypotension (compensated CS), inotropes (dobutamine or levosimendan) may be cautiously started after first the bolus of fluids has failed to restore peripheral organ perfusion. Attention is required in patients with RV dysfunction who may not tolerate fluid administration or in patients with signs of congestion. When hypotension is also present, concomitant administration of an inotropic agent and a vasopressor (preferably norepinephrine) should be initiated and titrated until perfusion is restored. In patients with refractory shock, escalation of therapy is required with the addition of a second vasopressor (vasopressin) or MCS [99].

Norepinephrine is recommended as a first-line vasopressor (Class IIb/B recommendation) [4] to restore end-organ perfusion and maintain systolic blood pressure [1,97]. Additionally, through cardiac β1 adrenergic stimulation, it enhances cardiac contractility and ventricular–arterial coupling [100]. The exact target of SBP is not fully clarified, but an initial goal of SBP > 90 mmHg and/or MAP of 55–75 mmHg in conjunction with other clinical markers of end-organ perfusion is advised [54]. Compared to epinephrine, norepinephrine had similar hemodynamic effects on mean arterial pressure (MAP) and CI, but epinephrine was associated with higher rates of refractory shock, tachycardia, lactic acidosis [101] and mortality [102,103]. Moreover, compared to dopamine, norepinephrine had a safer profile in patients with CS, due to a lower trend for arrhythmic events and mortality [104]. Vasopressin lacks inotropic properties and may be used as a second-line vasoactive agent, concomitantly to norepinephrine, if hemodynamic status does not improve with single use of norepinephrine [1,99,105]. Its administration may be appealing in special circumstances, like in patients with right ventricular failure, as it does not affect pulmonary arterial pressure [93,97,106], or in combination with milrinone in order to counteract its vasodilatory effect [1], but evidence is lacking [93].

Inotropes may be considered in addition to vasopressors in order to augment cardiac output and end-organ perfusion (Class IIb/C recommendation) [4]. Dobutamine is recommended over other inotropic agents if signs of hypoperfusion persist despite first-line vasopressor therapy [1]. However, a systematic review failed to show any benefit of dobutamine over levosimendan for short- and long-term survival [107]. Levosimendan and milrinone may be preferable over dobutamine in special cases such as long b-blockade, right ventricular dysfunction, pulmonary hypertension, or Takotsubo cardiomyopathy [1,4,108].

In CS patients with refractory hypotension, vasopressin may aid in preserving arterial blood pressure, as a third vasoactive agent, in conjunction with norepinephrine and dobutamine [99].

### 5.5. Short-Term Mechanical Circulatory Support

Patients who present with deteriorating or extremis CS, or those who fail to stabilize hemodynamically with two vasoactive agents may benefit from devices for temporary MCS in an individualized manner (Class IIa/C recommendation) [4,22]. Early initiation of MCS may provide univentricular or biventricular support by improving cardiac contractility, reducing left/right ventricular end-diastolic pressures, enhancing coronary perfusion and decreasing myocardial oxygen demand [109,110], resulting in effective weaning from cardiotoxic vasoactive agents [111]. However, controversial results with respect to mortality [112,113,114,115] incite the consideration of challenging issues regarding their application in practice, such as patient selection, type of device, appropriate timing, and prognostic impact. A characteristic recent example is the ECMO-CS randomized controlled trial where patients with deteriorating CS were randomized to immediate ECMO or early conservative therapy (with the ECMO kept as a bailout option at a later stage). The study did not show a benefit for the early ECMO approach. Despite having several limitations (i.e., relatively small sample size, mixed cohort of AMI- and non-AMI-related CS, and significant crossover rate (39% crossover to VA-ECMO in early conservative arm)), the study provides important data for the CS population.

Available choices for left ventricular assistance include the intra-aortic balloon pump (IABP), microaxial flow pumps (Impella CP, Impella 5), or the left-atrium-to-femoral-artery system device (Tandem Heart), while right ventricular assistance may be supported by the Impella RP, Tandem Heart RA-PA and Protek Duo devices. Venous–arterial extracorporeal membrane oxygenation (VA-ECMO) may reinforce biventricular performance and improve oxygenation [1,55,110]. Regarding the down-side of advanced percutaneous left ventricular devices, IABP use has subsided, taking into account no proven survival benefit for patients with AMI-CS [112], and currently its implementation may be considered for patients with refractory CS of non-AMI etiology (class IIb/C recommendation) [4] or for AMI-CS patients with mechanical complications as a bridge to more advanced MCS devices (class IIa/C recommendation) [42]. Even if Impella devices seem like promising approaches, data regarding their beneficial effect on mortality are scarce [113,114,116]. Although ECMO may ensure hemodynamic stabilization in cardiopulmonary resuscitation [117], it may also increase LV afterload, making its use reasonable with concomitant LV unloading (IABP, Impella, septostomy and hybrid circuit configuration) [1,110,118]. It must be emphasized that devices like Impella and ECMO necessitate the insertion of large-bore cannulas into major vessels and carry a high risk of complications, including vascular and bleeding complications. Characteristically, for Impella used in AMI-related CS, the rate of severe bleeding reported in the literature ranged from 8.5% to 31% [119,120]. Thus, to improve the efficacy of advanced MCS devices and increase the chances of positive studies in the CS field, specific measures should be taken to reduce complications. Such measures include comprehensive training in device insertion and maintenance, formation of dedicated teams (e.g., ECMO team including perfusionists), ultrasound guided vascular access, use of vascular closure systems, etc.

The uniqueness of each device, indicated by distinct hemodynamic effects, favorable profiles, contraindications and complications, limits their use to selected patients and under the supervision of expert teams [121]. The comprehensive approach by multidisciplinary teams in the context of a shock network emerges as an ultimate but not impossible goal to improve survival. Interestingly, recent data support that early initiation of MCS in the initial stages of CS, even before the administration of inotropes or the PCI strategy, is significantly associated with increased survival rates in patients presenting with AMI-CS [122], supporting the need to achieve shorter “door to support” intervals, so as to anticipate the deleterious effects of the fatal spiral of cardiac compromise [123].

## 6. Shock Teams and Networks

The holistic management of CS, which includes early identification, comprehensive diagnostic work-up and treatment interventions, requires a multidisciplinary approach. The variety of CS presentations, severity and causes, the lack of strong evidence for proposed treatments (e.g., t-MCS), and the need for an individualized therapeutic approach make collective decision making and pooling of specialized expertise even more important. There is increasing evidence that the introduction of dedicated shock teams and networks could improve clinical outcomes in CS patients.

The introduction of the hub-and-spoke model has a positive impact on patient outcomes [124,125,126]. The “hub” hospital has the central role, warranting the presence of a multidisciplinary CS team consisting of the interventional cardiologist, critical care specialist, cardiothoracic surgeon and advanced heart failure specialist (Level of care I). The “spoke” hospitals are either PCI-capable centers without advanced MCS (Level of care II) or non-PCI-capable hospitals, both referring to the hub hospital [121,127]. The role of the shock team is the timely recognition of CS and the provision of appropriate consultation to spoke hospitals regarding escalation of therapy and the need for advanced MCS, right heart catheterization (RHC) for the employment of the appropriate MCS modality, initiation of MCS, advanced postintervention care and monitoring, and eventually weaning from the MCS device and patient recovery [11,127]. Moreover, Rab comments on the fundamental role of a “shock doc”, a distinct member of the shock team who coordinates patient pathways and is responsible for the synchronization of intervention strategies [128]. Implementation of regionalized shock protocols in both AMI-CS and non-AMI-CS populations has proven to be feasible and was associated with improved survival [34,69,129,130]. A multicenter observational study, which compared CICUs with or without shock teams, concluded that the presence of shock teams was linked to more extensive use of pulmonary catheter catheterization and decreased administration of vasoactive agents. Furthermore, centers with a shock team used less MCS overall, but when MCS was used, it more often involved an advanced device (e.g., IMPELLA, ECMO or Tandem Heart). Mortality was substantially lower (23% versus 29%; adjusted OR: 0,72; 95% CI: 0.55–0.94; *p* = 0.016), as was the proportion of patients necessitating renal replacement therapy or mechanical ventilation, depicting a moderate degree of end-organ damage [131].

In this model, the ED has a strategic role outlined by the early identification of CS patients, initial stabilization, and prompt triage to the appropriate level of care [127]. Emergency physicians should endorse unified shock protocols in alliance with the hub hospital and activate the shock team through a one-call line for multidisciplinary consultation. The importance of expedient triage is underscored by an observational retrospective study that reviewed CS patient admissions from the ED directly to a CICU or an ICU. Patient admission to a non-CICU was associated with significant treatment delays for disease-modifying and evidenced-based therapies like RHC, PCI or MCS, and eventually higher mortality [132]. Therefore, direct transfer of CS patients to the spoke hospital is a priority in order to guarantee optimal management without delays.

Criteria for shock team activation vary among different shock team initiatives that implement regional multidisciplinary strategies. Prespecified inclusion criteria in the Detroit Cardiogenic Shock Initiative comprise AMI (indicated by ischemic symptoms/ECG and/or biomarker investigations) or CS (indicated by hypotension < 90/60 mmHg or the need for vasopressors/inotropes to maintain SBP > 90 mmHg) and evidence of hypoperfusion (cool extremities, oliguria, lactic acidosis) that settle the activation of the catheterization lab, in the absence of any exclusion criteria, where hemodynamic criteria will tailor further management [34]. The INOVA pathway and the Utah Cardiac Recovery Shock Team propose the activation of the shock team upon even the suspicion of CS with clinical criteria (SBP, evidence of end-organ perfusion, lactate) [69,130]. Finally, the University of Ottawa Heart Institute recommends that upon clinical identification of CS, the coronary cardiac unit senior resident has the key role in assessing the patient for possible exclusion criteria and successively activating “CODE SHOCK” [129]. Thus, it seems that clinical evaluation is the mainstay for setting the alarm code.

**Figure 1:**
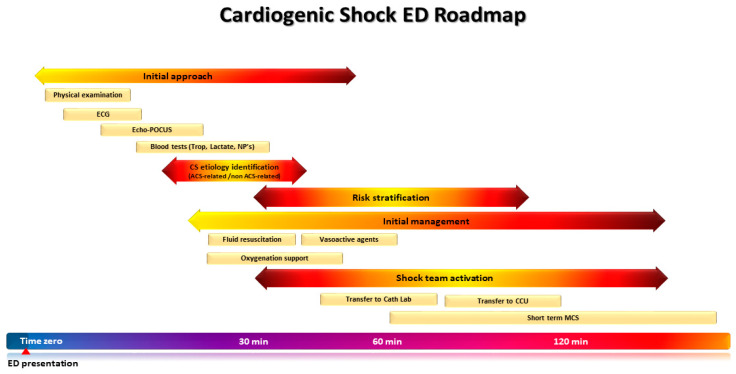
The roadmap of the patients presenting in the emergency department (ED) with cardiogenic shock (CS). A timely and structured approach upon presentation to the ED ensures prompt identification of CS, its underlying etiology, and risk stratification, in order to guide decision making and patient disposition without delay.

The roadmap of the patient presenting to the ED with CS is summarized in Figure 1.

## 7. Conclusions

The battle against CS necessitates a deep insight into the complexity of the syndrome and a prompt multidisciplinary approach. Current trends in epidemiology and a more nuanced stage classification may help clarify the broad spectrum of clinical phenotypes and optimize individualized management according the the culprit cardiac insult. Prompt identification and continuous assessment of CS patients are the foundations of the appropriate treatment plan. Critical evaluation in the acute setting should be directly correlated with the activation of a medical “defense cascade”, which in turn should guarantee high quality interventions. The ED, which occupies a crucial position, in alliance with the fundamental performance of the shock team, is a promising bundle for the improvement of CS mortality. Attention should not only be driven towards the development of proficient MCS devices but also to the adoption of readily available diagnostic tools and decision-making algorithms that will ensure expedient patient direction through the CS network. The establishment of validated risk stratification scores, the determination of uniform and readily available clinical criteria for shock team activation, and the endorsement of precise exclusion criteria are special issues to be addressed. Thus, further clinical studies should be organized on this basis, so as to facilitate optimal CS patient management.

## Figures and Tables

**Table 1:** Definition of Cardiogenic Shock in various clinical trials and guidelines.

SHOCK (1999) [13]	CARDSHOCK (2015) [8]	SCAI (2019) [22]					ESC (2021) [4]
		A—At Risk	B—Beginning	C—Classic	D—Deteriorating	E—Extremis	
		Clinical Criteria					
ACS cause Clinical criteria: a. SBP < 90 mmHg for at least 30 min to maintain an S/need for supportive measures, BP ≥ 90 mm Hg and b. end-organ hypoperfusion (cool extremities or UO < 30 mL per hour, and HR ≥ 60 bpm)	Cardiac cause and a. SBP < 90 mmHg (after adequate fluid challenge) for 30 min/need for vasopressor therapy to maintain SBP > 90 mmHg and b. signs of hypoperfusion (altered mental status/confusion, cold periphery, oliguria < 0.5 mL/kg/h for the previous 6 h, or lactate > 2)	SBP ≥ 100 mm Hg	a. SBP < 90 or MAP < 60 or >30 mmHg drop from baseline b. HR ≥ 100	SBP < 90 or MAP < 60 or >30 mmHg drop from baseline and drugs/MCS to maintain SBP > 90 mmHg	any of stage C and requiring multiple pressors OR addition of MCS to maintain perfusion	- No SBP - PEA or refractory VT/VF - BP < 90 mmHg despite maximal support	Cardiac cause and a. clinical signs of hypoperfusion (cold sweated extremities, oliguria, mental confusion, dizziness, narrow PP) b. biochemical manifestations of hypoperfusion (creatinine ↑, metabolic acidosis, lactate ↑)
		Biochemical markers					
		Normal creatinine, lactate	Lactate < 2, creatinine ↑/-, BNP ↑	-lactate > 2 -creatinine × 2 -LFT, BNP ↑	Any of Stage C and deteriorating	- CPR (A-modifier) - pH ≤ 7.2 - lactate ≥ 5	
Hemodynamic criteria: a. CI ≤ 2.2 L/min/m^2^ and b. PCWP ≥ 15 mm Hg		Physical exam					
		Normal JVP, clear lung sounds, strong distal pulses, normal mentation	↑ JVP, rales, strong distal pulses, normal mentation	↑ JVP, rales, unwell/agitated, cold, clammy extremities, UO < 30 mL/h	Any of stage C	Cardiac collapse	

Abbreviations: ACS = acute coronary syndrome; CI = cardiac index; CPR = cardiopulmonary resuscitation; HR = heart rate; JVP = jugular venous pressure; MAP = mean arterial pressure; MCS = mechanical circulatory support; LFT = liver function tests; BNP = B-Type Natriuretic Peptide; PCWP = pulmonary capillary wedge pressure; PEA = pulseless electrical activity; PP = pulse pressure; SBP = systolic blood pressure; UO = urine output; VF = ventricular fibrillation; VT = ventricular tachycardia, ↑ = increased, - = no difference

**Table 2:** Useful risk scores for the risk stratification of cardiogenic shock (CS) patients. Risk scores highlighted with * could be used in the Emergency setting.

Score, Year	Number of Patients, Population	Risk Assessment, Patient Classification	AUC	Criteria								Limitations/Validation
				History	Physical Exam	Vital Signs	Labs	Invasive	Imaging	Therapy	Other	
CardShock *, 2015 [8]	N = 219/ACS-CS, non-ACS-CS	In-hospital mortality Low risk—9% Moderate risk—36% High risk—77%	0.85	Age > 75 MI or CABG	Confusion		Lactate eGFR		LVEF < 40%		ACS etiology	external validation
IABP—Shock II, 2017 [68]	N = 600/ACS-CS	30 d mortality prediction Low risk—23.8% Moderate risk—49.2% High risk—76.6%		Age > 73 Stroke			Creatinine Glucose Lactate	TIMI flow < 3 after PCI				Only ACS-CS patients. External validation
CLIP score * [75]	N = 458/ACS-CS	30 d mortality prediction Linear model	0.82 c-statistic/AUC 0.79				Cystatin Lactate IL-6 NT-proBNP					Only ACS-CS patients, external validation
CSP, 2022 [72]	N = 311/CS	In-hospital mortality Low risk—0% Moderate risk—8.75% High risk—66.7%	0.941	CAD			Hb		LVEF > 40%	CPR time Albumin RRT > 1 Inotropes		-Diseased patients in <24 h were excluded -no external validation
SCAI *, 2019 [22]	Based on expert consensus	Shock severity			Mentation JVP Lung auscultation Skin perfusion Volume overload	SBP, HR	Creatinine Lactate LFTs pH	If available: CI PCWP PA saturation		CPR Multiple Inotropes MCS NIV/IMV Defibrillation		External validation
IHVI CS risk score, 2019 [69]	N = 204 /ACS-CS, non-ACS-CS	30 d mortality prediction Low risk—0% Moderate risk—17.6% High risk—82.4%	0.97	Age ≥ 71 DM			Lactate	CPO PAPi		Inotropes > 36 h RRT		Comparison only with CardShock score
ORBI, 2018 [66]	N = 6838	Prediction of development of CS Low risk—1.3% Low to Intermediate risk—6.6% Intermediate to High risk—11.7% High risk—31.8%	0.80 C-statistic	Age > 70 Stroke, TIA	Killip class on admission.	SBP, PP, HR	Glucose	Culprit lesion of the LMCA. TIMI flow < 3 after PCI			Cardiac arrest on presentation. Anterior MI. FMC-PCI delay > 90 min	External validation
Henning et al. *, 2018 [74]	N = 700/hypotensive patients in the ED	Prediction of cardiogenic hypotension ≥ 2, cardiogenic hypotension	0.83	HF	Shortness of breath	Absence of fever	troponin				ECG findings of ischemia	No external validation
Chang et al. *, 2022 [73]	N = 5.881/patients who developed CS	Prediction of development of CS machine learning model based on the XGBoost algorithm	0.87	Age Male gender		Temperature PP SpO2	Troponin Glucose Immature granulocytes bicarbonate					No external validation

Abbreviations: ACS = acute coronary syndrome; ACS-CS = cardiogenic shock associated with acute coronary syndrome; CABG = coronary artery bypass graft; CAD = coronary artery disease; CI = cardiac index; CPO = cardiac power output; CPR = cardiopulmonary resuscitation; CS = cardiogenic shock; DM = diabetes mellitus; ECG = electrocardiogram; eGFR = estimated glomerular filtration rate; FMC = first medical contact; Hb = hemoglobin; HF = heart failure; HR = heart rate; IL-6 = intereleukin-6; IMV = invasive mechanical ventilation; JVP = jugular venous pressure; LMVA = left main coronary artery; LVEF = left ventricular ejection fraction; MCS = mechanical circulatory support; MI = myocardial infraction; LFT = liver function tests; NIV = noninvasive ventilation; NT-proBNP = N-Terminal Pro–B-Type Natriuretic Peptide; PA = pulmonary artery; PCI = percutaneous coronary intervention; PAPi = pulmonary artery pulsatility index; PCWP = pulmonary capillary wedge pressure; PP = pulse pressure; RRT = renal replacement therapy; SBP = systolic blood pressure; SpO2 = capillary oxygen saturation; TIMI = thrombolysis in myocardial infarction.

**Table 3:** Indications and initial settings for both noninvasive ventilation (NIV) and invasive mechanical ventilation (IMV) [89,90].

Mechanical Ventilation			
	Noninvasive Ventilation		Intubation and Invasive Ventilation
	C-PAP	Bi-PAP	Intubation
Indications	Hypoxia without hypercapnia	Hypoxia + hypercapnia	Failed NIV trial Refractory shock Altered mental status Severe acid–base disorder Scheduled PCI and concomitant respiratory failure
Initial Settings	Initial Pressure: 5–10 cm H_2_O Increase by 2 cm H_2_O to target oxygenation Target SPO_2_ ≥ 92%	Initial IPAP: 10 cm H_2_O Increase by 2 cm H_2_O up to 20 cm H_2_O Avoid IPAP > 20 cm H_2_O Initial EPAP: 5 cm H_2_O Increase by 2 cm H_2_O to target oxygenation	**MV in non-RV failure patients**
			Initial FiO_2_ = 100%, V_T_ = 6–10 mL/kg of IBW, RR = 10–20 breaths/min Maintain Ppl < 30 cm H_2_O, monitor auto-PEEP PEEP = 5–10 cm H_2_O, Increase by 2 cm H_2_O to target SPO_2_
			**MV in RV failure patients**
			Avoid PPV if possible Optimize hemodynamics Maintain normovolemia Induction agents of choice: Ketamine (1–2 mg/kg) or Etomidate (0.1–0.3 mg/kg) Opioids Benzodiazepines Avoid Propofol Administer all induction agents at lowest necessary dose Drug combinations may be preferable Initial PEEP 2–5 cm H_2_O—maintain low PEEP

Abbreviations: Bi-PAP = bilevel positive airway pressure; C-PAP = continuous positive airway pressure; EPAP = expiratory positive airway pressure; FiO2 = fraction of inspired oxygen; IBW = ideal body weight; IPAP = inspiratory positive airway pressure; MV = mechanical ventilation; NIV = noninvasive ventilation; PCI = percutaneous coronary intervention; PEEP = positive end-expiratory pressure; Ppl = plateau pressure; PPV = positive pressure ventilation; RR = respiratory rate; RV = right ventricular; SPO_2_ = peripheral capillary oxygen saturation; VT = tidal volume.

## Data Availability

No new data were created or analyzed in this study. Data sharing does not apply to this article.
